# Open Doors by Fair Means: a quasi-experimental controlled study on the effects of an open-door policy on acute psychiatric wards

**DOI:** 10.1186/s12913-022-08322-6

**Published:** 2022-07-22

**Authors:** Lisa K. Schreiber, Florian G. Metzger, Erich Flammer, Heike Rinke, Andreas J. Fallgatter, Tilman Steinert

**Affiliations:** 1 University Department of Psychiatry and Psychotherapy Tuebingen, Calwerstr. 14, 72076 Tuebingen, Germany; 2 Geriatric Center, University Hospital of Tuebingen, Calwerstr. 14, 72076 Tuebingen, Germany; 3 Vitos Hospital for Psychiatry and Psychotherapy Haina, Landgraf-Philipp-Platz 3, 35066 Haina (Kloster), Germany; 4 Centers for Psychiatry Suedwuerttemberg, Ulm University, Weingartshofer Str. 2, 88214 Ravensburg-WeissenauRavensburg, Germany

**Keywords:** Safety, Ward, Psychiatry, Absconding, Coercive measures, Compulsory treatment, Restraint, Absconding, Open door policy, Ward climate, Ward atmosphere, Suicide

## Abstract

**Background:**

Psychiatric wards treating involuntarily admitted patients are traditionally locked to prevent absconding. However, on the basis of observational evidence, the necessity for locked units in psychiatric hospitals has increasingly been questioned. Updated Mental Health Laws in several Federal States of Germany legitimate involuntary commitment without generally locked doors.

**Methods:**

We examined the effects of an open-door policy in a quasi-experimental, prospective design. For the first time, at each of two locations, two identical wards serving as control and intervention could be compared. After a baseline period of three months, one ward at each location started the 12 month intervention period with the implementation of an open-door policy, while the respective control ward, as before, used open doors only facultatively. Primary outcomes were average opening times of the four wards between 8 a.m. and 8 p.m., and the number of involuntary treatment days with the doors open. Secondary outcomes were adverse events including aggressive incidents, absconding, suicide attempts and coercive measures.

**Results:**

Overall, door-opening times increased significantly at both sites´ intervention wards. The number of adverse events did not increase during intervention period. Frequencies of coercive measures decreased in Friedrichshafen and remained unchanged in Tuebingen. In case of the intervention ward in Friedrichshafen, doors were open in up to 91% of all involuntary treatment days, whereas in the control ward, this was only the case in 67% of all involuntary treatment days (*p* < .001). In case of the intervention ward in Tuebingen, 45% of involuntary treatment days had open doors, compared to 30% in the control ward (*p* < .001).

**Conclusions:**

It is possible to manage psychiatric wards with open doors without taking inappropriate risks. The extent to which open-door policies are achievable is be dependent on staffing and patient characteristics. Further research is necessary to explore the role of staff attitudes.

**Trial registration:**

Our trial "Open Doors by Fair Means" is retrospectively registered with DRKS (DRKS00015154) on Sept. 10^th^ 2018 and displayed on the public web site. It is searchable via its meta-registry (http://apps.who.int/trialsearch/).

## Introduction

Psychiatric wards with involuntarily hospitalized patients due to endangering themselves or others are usually locked to prevent absconding. Compared to other coercive measures such as seclusion or restraint, a closed door appears to be restricting personal freedom less. However, locked doors have considerable negative impact in terms of stigma and ward atmosphere [1]. Moreover, locked doors are often hard to justify as only a minority of patients is actually prone to absconding. For this reason, in Austria it is prohibited to treat voluntary patients on wards with locked doors. As a consequence, locked wards are nearly non-existent there [2]. In Germany, approximately 10% of the patients being treated in psychiatric acute care units, most of them locked, stay there involuntarily due to a court’s decision [3]. The number has even increased over the past years [4]. Although locked doors are often justified by the fact that involuntarily committed patients should be prevented from leaving the ward, doors in most countries usually remain locked even when no patient is being committed involuntarily [5].

In a Swedish study, the authors found that 19% of the wards were locked without a patient being committed involuntarily. Conversely, in case of 19% of the wards that were stated to be open at least one patient was committed involuntarily [5]. Similar to most other developed countries, the preconditions of involuntary commitment in Germany are the presence of a mental illness as well as the risk of imminent personal injury to self or others and mental aberration. Similar to most other developed countries, the preconditions of involuntary commitment in Germany are the presence of a mental illness as well as the risk of danger to oneself or others and causation by the respective mental disorder. All decisions on involuntary commitment must be made by a court. Only in acute cases patients may be detained preliminarily until the end of the day after admission. The Mental Health Law for the Federal State of Baden-Wuerttemberg provides detailed regulations for the use of any coercive measures. According to clinical judgement, unaccompanied leave is possible for patients who are treated involuntarily, too. Hence, the legal conclusion is drawn in practice that psychiatric wards must not be generally locked, if absconding can be prevented otherwise [6]. This development of legislations, that is in accordance with demands of service user organizations, psychiatric societies, and the UN Convention on the Rights of Persons with Disabilities, causes an urgent need for sound empirical research. Up to now, there is preliminary evidence from several cross-sectional and pre-post studies, predominantly from Switzerland [7, 8] and Germany [9, 10], that we had reviewed in a previous paper [11]. These studies suggest that suicide attempts, suicides, violent acts, and absconding are not more frequent in wards with an open-door policy.

Within recent years, observational evidence suggested that wards with an explicit open-door policy did not perform worse than locked doors in terms of preventing suicides and violent acts. However, fairness of comparisons and selection bias have been an issue of discussion [12]. Nevertheless, since 2015, several updated Mental Health Laws of Federal States in Germany basically enable treatment of involuntarily committed patients on open wards, if absconding is prevented by other appropriate means. For instance, the Mental Health Law of North Rhine-Westphalia states that”hospitals have to ensure by appropriate measures that the patients concerned do not withdraw themselves from the commitment” [13]. In a large observational study, data reported from 21 German hospitals with almost 350,000 cases over the course of 15 years found that suicides and suicide attempts, as well as absconding, did not occur more frequently in wards with open doors [7]. Limiting the conclusions which can be drawn, it has to be noted that data was collected retrospectively and the definition of open, semi-open and locked wards was fairly imprecise. Further doubts remain whether the definition of ‘open-door policy’ was precise enough, ranging from an informally expressed attitude to objectively open doors for 24 h on 365 days [11]. Prospective controlled trials with previously defined outcomes are missing.

The reason for the widespread use of locked wards may be due to treatment culture, control attempts and uncertainties [4, 14], but is by no means a therapeutic intervention. It relates primarily to the fear of involuntarily treated patients absconding and at worst committing suicide or harming themselves or others. However, between 50–85% of all suicides by psychiatric in-patients occur during a permitted leave [15]. Also, with regard to aggressive incidents and absconding, previous findings hint on the possibility that the opposite could be true: locked doors might increase the risk of absconding, of violent conflicts between patients or between patients and staff and further might reduce treatment compliance [9, 16].

### Aims of this study

The present study investigates the effects of an open-door policy on psychiatric acute care units. At two different locations, two identical wards in terms of staffing, architecture, admissions and treatment setting serving as control and intervention could be compared. After a baseline period of three months, one ward at each location started the 12 month interventions period with the implementation of an open-door policy, while the respective control ward, as before, used open doors only facultatively. A comprehensive presentation of the research project has been published as a study protocol [17]. The real challenge to manage psychiatric wards with open doors is eventually to keep doors open with involuntary treated patients present. As reported in other studies [1, 7–10], an open-door policy means that ward entrance doors are generally kept open during daytime. Locking the doors is considered as an exception that needs good reasoning and subsequent reviewing whether it is still necessary. However, open doors are not considered as an absolute requirement, inappropriate risks for patients and others must be avoided. Accordingly, we determined the average opening time between 8 a.m. and 8 p.m. in percent, and the number of days of involuntary treatment with open doors as primary outcomes. Secondary outcomes were the frequency of adverse events such as absconding, suicide attempts or aggressive incidents, and frequency of coercive measures.

## Methods

### Study design

Here, we present quantitative data of the intervention period. Follow-ups and qualitative results of the mixed-methods design are not part of this paper. A detailed description of the entire project can be found in the study protocol [17].

In the present study, we investigated the effects of an open-door policy on acute care wards that have been declared as ‘optionally open ‘ before but were actually mostly locked. More specifically, we compared two intervention wards and two control wards at two different sites, Tuebingen and Friedrichshafen, in the federal State of Baden-Wuerttemberg, Germany. The study was carried out between June 2017 and September 2018 starting with a three-month baseline period to assess the corresponding outcome measures without any intervention. The staff remained assigned to the individual wards during all study periods.

In a strictly alternating manner, patients were admitted to one of the two wards of each site which are identical in their architecture. This enabled a quasi-experimental design. If the number of patients differed by more than three, the strict alternation could be paused.

While business as usual ran on the control wards, interventions were implemented on the intervention wards, where staff should try to realize open doors with all reasonable efforts, but not at any prize and without taking foreseeable risks.

Before the start of the project, the employees of each participating ward were informed about the content of the research project, as well as about the background and current research findings. The patients were regularly informed as part of the morning round. Here the information was limited to the fact that the doors could be open or closed and that they preferably should let the staff know if they wanted to leave the ward.

Ethical approval was obtained from the ethics committees of the universities of Tuebingen on June 6^th^, 2017, No. 170/2017/BO1 and Ulm on March 1st, 2017, No. 313/16, respectively.

### Locations

The wards in Friedrichshafen and Tuebingen both belong to a psychiatric hospital that is responsible for a specific catchment area in the federal State of Baden-Wuerttemberg. Both hospitals provide many aspects of specialized in-patient care such as diagnosis-specific treatment programs, e.g. for psychotic and affective disorders, trauma and borderline personality disorder, accompanied by medical, psychotherapeutic and occupational therapy offers. On the admission wards for general psychiatry which participated in this study, mostly patients with severe mental disorders are admitted, with 17% of the patients being hospitalized involuntarily.

### Participants

Overall, 3,270 admissions were recorded during the survey period between June 2017 and August 2018, 1,495 in Friedrichshafen and 1,775 in Tuebingen. Patients were on average 45.7 years old (SD = 17.21), with a somewhat higher average age of 46,3 in Friedrichshafen than in Tuebingen (45,2), but without differences between sites or each site´s control and intervention ward (H(3) = 5.247, *p* = 0.155). 50% of all patients were female. The distribution of the primary diagnoses is shown in Table 1.

**Table 1:** The psychiatric diagnoses according to ICD-10 presented by people hospitalized in intervention and control wards

	Ward	% Diagnoses					
		F0	F1	F2	F3	F4	F6
Control	FNC	8.1	3.4	49.0	23.3	7.5	8.2
Intervention	FNI	5.3	5.6	43.3	20.8	13.3	11.3
Control	TUC	2.7	6.8	56.4	17.1	4.7	8.9
Intervention	TUI	8.1	6.1	29.4	30.5	9.9	16.0

*Note*. Top: FNI = Friedrichshafen intervention ward, FNC = Friedrichshafen control ward. Bottom: TUI = Tuebingen intervention ward, TUC = Tuebingen control ward. Please note that the distribution of diagnoses does differ significantly between each sites´ control and intervention wards (*p* < 0.001). Diagnoses according to ICD-10: F0 = Organic, including symptomatic, disorders; F1 = Mental and behavioral disorders due to psychoactive substance use; F2 = Schizophrenia, schizotypal and delusional disorders; F3 = Mood (affective) disorders; F4 = Neurotic, stress-related and somatoform disorders; F6 = Disorders od adult personality and behavior

### Interventions

The core intervention was a change in the team´s mindset: Between 8 a.m. and 8 p.m., doors remained open and should only be closed due to specific reasons, the presence of which were to be decided in a team meeting. In order to implement this, staff were fully informed about the project and introduced to the interventions before the start of the project. Patients were informed about the open-door policy at admission.

The treatment team was asked to discuss possible obstacles at the beginning of each shift change at 8 a.m. and 1 p.m. and to decide whether doors could stay open or should be closed. In this context, it was also possible to identify patients at risk and to plan appropriate interventions, e.g. more intensive care, accompanied leave, activities or a nurse sitting near the entrance door to offer company or supportive talks. Weekly staff meetings took place to reflect on difficult situations, concerns and strategies. Closing the wards was possible at any time.

In case of the Friedrichshafen intervention ward, an additional nurse was hired to enable more 1:1 interactions with patients. On the Tuebingen intervention ward, interns and nursing students were involved in the implementation of interventions.

At a glance, interventions were [17]:

- The door status was discussed each morning with the complete staffing team (doctors and nurses). Reasons requiring a locked door were documented and individual interventions for patients at risk were planned (e.g. accompanied leave, planned visits at home, activities on or outside the wards as well as therapeutic and deescalative talks)
- Weekly team meetings to discuss special events or concerns
- An additional nurse for the ward team was deployed in Friedrichshafen; in Tuebingen, nursing trainees were involved in taking care of patients in need of support
- The “Potsdam Table” is a small sitting area with a nurse as a contact person next to the ward door, providing a meeting facility that might dissuade endangered patients urging to leave the ward by deescalating conversations. The contact person can respond by offering contact, initiating activities, and in case of doubt also deciding that the door should be closed.

### Standard care

The control wards continued to be opened facultatively without additional interventions.’Facultatively’ means that the ward may be unlocked if possible, but no special efforts are undertaken in ward policy to do so. As a consequence of this long-standing policy on all participating wards, an open entrance door on an admission ward was not a new challenging event per se, neither for staff nor patients.

For several years, staff members on all participating wards have had the opportunity to take part in de-escalation training courses at regular intervals. This offer remained unchanged during the survey period.

### Outcome measures

The primary outcome measures were the average opening time of the four acute wards between 8 a.m. and 8 p.m. in percent, as well as the number of involuntary treatment days with the door open. Each patient being treated involuntarily on a specific day counts as one involuntary treatment day, e.g. we counted ten involuntary treatment days on one day, if ten patients were being treated involuntarily on that day. Door opening times were defined as percentage of the 12-h interval between 8 a.m. and 8 p.m. and counted in minutes, rounded to quarter steps of an hour. For instance, if the door was open from 9 a.m. until 10.20 a.m., we documented 1.25 of 12 h which corresponds to 10.4%. As secondary outcomes, the frequency of aggressive incidents including severe self-harm, absconding, suicides, and suicide attempts were recorded as well as the use of coercive measures such as seclusion or restraint.

### Statistical analysis

Analysis was performed using IBM SPSS Statistics, version 27.0. As we found most of the data to be non-normally distributed, we performed Mann–Whitney-U tests to determine differences between two groups and Kruskal–Wallis analysis of variance with Dunn-Bonferroni post-hoc tests for more than two groups. Differences between pre-intervention period and post-intervention period were tested with Wilcoxon Signed Rank tests for paired groups. We used an alpha level of 0.05 for statistical analyses of sample characteristics. According to our three primary outcome measures, we used an adjusted alpha level of 0.015 for analyses of door opening times, number of treatment days and involuntary treatment days. For analyses of adverse events at the two sites, we used an alpha level of 0.025.

### Role of the funding source

The funders had no impact on the study design, data collection and analysis, decision to publish or preparation of the manuscript.

## Results

Table 2 presents the mean number of patients present per day and the mean number of admissions per day. Regarding the total number of patients, no significant differences were found between baseline (Mdn = 19) and intervention period (Mdn = 19), U = 255,971.5, *p* = 0.160 nor between control (Mdn = 19) and intervention group (Mdn = 20), U = 436,127.5, *p* = 0.101. Regarding the number of daily admissions, no significant differences were found between baseline (Mdn = 1) and intervention period (Mdn = 1), U = 271,620.0, *p* = 0·671 nor between control (Mdn = 1) an intervention group (Mdn = 1), U = 421,167.0, *p* = 0.627. In summary, no significant differences between intervention and control wards were found, suggesting that the alternating allocation of admissions between the wards was successful in this aspect.

**Table 2:** Number of patients and admissions per day during baseline and intervention period

	Ward	Number of patients per day				Admissions per day			
		M	SD	Mdn	IQR	M	SD	Mdn	IQR
Baseline	FNC	24.96	1.26	24.0	1.25	0.84	0.86	2.0	1.25
	FNI	25.43	1.49	30.0	3.0	1.07	1.13	1.0	1.25
	TUC	16.39	1.98	19.0	5.0	1.73	1.35	1.0	1.0
	TUI	15.93	1.92	17.5	2.0	2.30	1.57	3.0	2.0
Intervention	FNC	22.47	1.78	23.5	3.5	1.10	1.02	1.0	1.0
	FNI	22.58	2.15	23.5	5.0	1.12	1.09	1.0	1.0
	TUC	17.16	1.51	16.5	3.0	1.93	1.47	2.0	2.0
	TUI	17.37	1.75	16.0	3.0	1.87	1.53	2.5	6.0

*Note*. *TUI* Tuebingen intervention ward, *TUC* Tuebingen control ward, *FNI* Friedrichshafen intervention ward, *FNC* Friedrichshafen control ward, *M* Mean, *SD* Standard deviation, *Mdn* Median, *IQR* Interquartile range

### Door opening times

All wards were open more frequently since the start of the project: The Friedrichshafen control ward was open 42.9% during baseline and 56.9% during intervention phase. The Friedrichshafen intervention ward was open 33.5% during baseline and 80.8% during intervention period. In Tuebingen, the control ward was open 0.3% during baseline and 17.8% during intervention period, while the intervention ward was open 21.5% during baseline and 30.7% during intervention period. A Kruskal–Wallis analysis of variance was conducted to test differences of door-opening times between control (Mdn = 0) and intervention wards (Mdn = 7) during intervention period. With H(7) = 530,704, *p* < 0.001, overall door opening times differed significantly. Comparing all pairs of wards, we found significant differences between the Friedrichshafen control and intervention ward (z = -7.85, *p* < 0.001), as well as between the Tuebingen control and intervention ward (z = -5.193, *p* < 0.001), which means that both sites´ intervention wards were open significantly longer than the control wards. Also, we found significant differences between baseline and intervention period within the Friedrichshafen control ward (z = -3.848, *p* < 0.001), as well as within the Tuebingen control ward (z = -3.169, *p* = 0.002).

### Voluntary and involuntary treatment days in relation to door status

Overall, we counted 36,657 treatment days (M = 20.05, SD = 3.60) at the four wards, out of which 6,235 (17.0%, M = 3.41, SD = 2.59) were involuntary. Across all wards and study phases, doors remained closed on 68 days even though none of the patients was treated involuntarily. To compare both sites´ proportion of involuntary treatment days, we divided the number of involuntary treatment days by the number of patients. Friedrichshafen (Mdn = 0.17) and Tuebingen (Mdn = 0.14) differed significantly (U(N_FN_ = 841, N_TU_ = 841) = 58,875.0, z = -5.25, *p* < 0.001). Absolute numbers and percentages of involuntary treatment days are shown in Table 3.

**Table 3:** Total number of treatment days and involuntary treatment days with related percentages of involuntary treatment days with open doors during baseline and intervention period

Period	Ward	Involuntary treatment days	% Involuntary treatment days with open doors	*p* Intervention vs. Control	*p* Baseline vs. Intervention
Baseline period	FNC	607	61.9		< 0.001
	FNI	495	23.2		0.004
	TUC	71	0		0.01
	TUI	329	16.1		0.28
Intervention period	FNC	1826	66.8	< 0.001	
	FNI	1161	90.7		
	TUC	804	30.1	< 0.001	
	TUI	1044	44.9		

*Note*. *TUI* Tuebingen intervention ward, *TUC* Tuebingen control ward, *FNI* Friedrichshafen intervention ward, *FNC* Friedrichshafen control ward

During intervention period in Friedrichshafen, doors were open in 66.8% of all involuntary treatment days in the control ward, while this was the case in 90.7% in case of the intervention ward (H(3) = 12.86, *p* < 0.001). In Tuebingen, doors were open in up to 30.1% of all involuntary treatment days on the control ward, while it was 44·9% in case of the intervention ward (H(3) = 4.30, *p* < 0.001).

### Reasons documented to keep the doors locked

During intervention period, reasons to keep the door locked were documented in 640 cases, relating to 468 different patients. The categories of reasons were not predefined, but only counted afterwards. In the majority of cases, a lack of communication skills due to illness (25.8%), suicidality (19.5%), and disorientation (12.9%) were given as specific reasons. Endangering others was recorded as a reason in 11.4% of the cases. Less frequently, specific symptoms of illness such as delusions and psychotic fears (6.3%), states of excitement (2.0%), and addiction-associated symptoms, e.g. intoxication or withdrawal (4.6%), were reported. In 3.1% of the cases, previous absconding was documented as a reason to keep the wards locked. Regarding structural aspects, the reported reasons “staff shortage”, “no doctor present” and “uncertainties on the part of physicians” sum up to a total of 2.5% of all cases.

The relative risk of a patient being documented as a reason for keeping the doors locked differed significantly between diagnoses. Patients with F2-diagnoses were disproportionately more often reported as patients at risk on the intervention wards (OR = 1.26), whereas patients with F3-diagnoses were the least likely to be documented as a reason (OR = 0.67).

### Safety: adverse events

The distribution of adverse events is displayed in Table 4. No clear pattern was observable. However, the frequency of adverse events was considerably different at baseline between wards, making it difficult to compare intervention and control wards during intervention period. A significant increase of adverse events from baseline (Mdn = 0) to intervention period (Mdn = 0) was observed at the Friedrichshafen control ward regarding coercive measures (z = 3.521, *p* < 0.001). Most adverse events decreased in intervention wards (z = -2.441, *p* = 0.015) and, to a descriptively smaller degree, in control wards (z = -1.721, *p* = 0.085). Due to multiple comparisons, we consider none of the observed changes as meaningful.

**Table 4:** Total number of adverse events per 1000 treatment days

	Aggressive incidents		Absconding		Suicide attempts		Coercive measures	
Ward	BL	INT	BL	INT	BL	INT	BL	INT
FNC	29.25	31.27	9.92	3.54	0.42	1.53	12.8	27.0
FNI	9.68	7.83	4.92	2.26	1.42	0.76	44.4	22.7
TUC	3.61	4.04	2.96	0.14	0.54	1.13	70.5	25.2
TUI	8.60	3.77	0.49	3.77	4.77	0.34	31.6	32.7

*Note*. *BL* Baseline, *INT* Intervention period, *FNC* Friedrichshafen control ward, *FNI* Friedrichshafen intervention ward, *TUC* Tuebingen control ward, *TUI* Tuebingen intervention ward

## Discussion

To our knowledge, this is the first prospective controlled trial with a quasi-experimental design for the implementation of an open-ward policy on acute care units. With over 3,000 treatment cases from two German psychiatric hospitals, our data is based on solid grounds. We managed to allocate the patients in a strictly alternating manner, ensuring the quasi-experimental design of the study. At both locations, mainly patients with psychotic or severe affective disorders were admitted to the acute care units. However, distributions of primary diagnoses differed significantly between each sites´ control and intervention wards. There is no clear explanation for the fact that particularly the number of patients with F2 diagnoses differ in this way. We assume that on the one hand the survey period was rather short and that the differences would have leveled out somewhat with a longer period of time. Even if we could not find any systematic evidence of this, it is possible that, especially outside the core working hours of the ward staff, i.e. in the evenings, at night and on weekends, or when several patients were admitted at the same time, patients were distributed more according to fit or custom. However, given the successful alternating allocation, this effect cannot yet be sufficient to explain this.

Regarding our primary outcomes, days with open doors increased 2.4-fold from 33.5% at baseline to 80.8% during intervention period in Friedrichshafen and 1.4-fold from 21.5 to 30.7% in Tuebingen with significant differences when compared to the control wards, respectively. The percentage of involuntary treatment days with open doors, which to our mind is the most robust indicator of an open-door policy, increased 3.9-fold from 23.2% at baseline to 90.7% during intervention period in Friedrichshafen and 1.9-fold from 16.1 to 30.1% in Tuebingen with significant differences when compared to the control wards. However, the control wards showed increased opening times, too, indicating potential spill-over effects between the wards. It might be possible that staff on control wards tried to demonstrate they could be as successful with open doors as their neighbored intervention wards.

Considerable differences between the sites at baseline make the interpretation of our findings more difficult but underscore the importance of conducting such a kind of study at more than one site. Baseline differences probably reflect longstanding attitudes and treatment practices within ward teams rather than differences between patient samples. As a matter of fact, the intervention was effective at both sites and there was no evidence of a ceiling effect, meaning that no further opening would have been possible in case of wards with a pre-existing open-ward policy.

However, even if the intervention was clearly effective, the intervention wards remained far from a 100% open-door practice which had been reported in the literature anecdotally [4, 11]. Reasons to keep doors locked were mostly patients with poor communication skills due to illness, disorientation, or acute suicidality. Establishing reliable relationships that allow agreements with those patients might be particularly difficult and staff for 1:1 supervision was not sufficiently available. Furthermore, some patients might consider continuous personal observation as more intrusive than a locked door. Consequently, we doubt that a 100% open-door policy can be realized on acute care wards with a considerable percentage of involuntary patients “by fair means”, i.e. without using other coercive interventions to prevent absconding. Differences between the two intervention wards show that criteria for locking the door in terms of safety reasons were differently specified, which cannot be explained sufficiently by differences of the patient sample. Probably not accidentally, the ward that was already more familiar with an open-door policy before the study achieved considerably higher rates of open doors. But, notably, this ward had received an additional nursing staff person for the time of the intervention, and for reasons beyond our control, no further nurse could be hired in Tuebingen. Instead, other assistants, of which the Tuebingen clinic as a teaching hospital has a larger number compared to Friedrichshafen, were involved. Although it is a topic of discussion that increasing the personnel ratio could contribute to reducing restrictive measures [18, 19], we cannot determine which single factors were the most relevant for a successful open-door policy.

One of the most important interventions at the patient level seems to be making individual arrangements that take into account the need for freedom and autonomy, including unaccompanied or accompanied leave in particular, but also more presence of staff in the entrance area and on the ward corridors [18, 19]. Furthermore, factors such as participation in decisions and acceptance of responsibility on the part of the patient are helpful to find a common understanding of the prerequisites of an open-door policy [20].

In terms of safety, suicides or violent attacks due to absconding did not occur during the data collection. We had registered aggressive incidents, suicide attempts, and absconding in the participating wards at baseline and during intervention period. Baseline data between wards differed significantly, which might be caused by single patients, considering the rather short period of three months. During intervention period, we did not observe a clear pattern of safety-relevant events. The number of adverse events mostly decreased on the intervention wards. Further, coercive measures, that were not allowed to be used to prevent absconding, occurred with roughly the same frequency in all participating wards during intervention period. That means that an open-door policy is not necessarily accompanied by more coercive interventions, as suggested by a recent study from four hospitals in Germany [21], but neither is there enough evidence to claim the opposite [11].

Our study has considerable strengths and some weaknesses. To our knowledge, this is the first study that investigated an open-door policy as an intervention in a prospective, controlled design on psychiatric wards treating involuntarily hospitalized patients. We did not only register the primary outcomes of open doors but also numerous other variables that can be considered as important control variables such as the percentage of treatment days with involuntary patients, absconding, suicide attempts, aggressive incidents, and the use of coercive measures. For the first time in the literature, we used a primary outcome, the percentage of involuntary treatment days with open doors, that we consider as the most conservative criterion of an open-door policy. Furthermore, a strength of this study is that it was conducted at two different sites. Like in many other studies with a small number of participating units, some of the results might be due to chance only and should not be interpreted as causal. For instance, absconding occurred more frequently in case of the intervention ward at one site but less frequent at the other. Moreover, the study at two sites allowed to compare the effects on wards with hospitals already applying open-door policies or not.

Our study has also several limitations. Whereas it included a detailed survey of some thousand admissions, the number of compared wards is relatively small. Robust evidence would require an RCT comprising dozens of hospitals which seems very difficult to achieve. Furthermore, our quasi-experimental design with alternating admissions on intervention wards and control wards had its limitations. From the presented data as well as from additional interviews, we observed considerable spill-over effects between the neighboring wards regarding the intervention. Staff on control wards started competing, resulting in an increase of open-door times also in case of the control wards. We found that the implementation of an open-door policy was successful, but we cannot determine which elements of the complex intervention were most effective. Notably, the ward that received an additional nursing staff position was particularly successful, but we do not know whether this was the reason. Eventually, we could not ascertain that secondary outcomes such as aggressive incidents were registered exactly in the same manner at both sites. Further studies on this issue are necessary.

## Conclusions

In this prospective study with a quasi-experimental design, we could show that it is possible to manage psychiatric wards with open doors without taking inappropriate risks. The extent to which an open-door policy can be implemented appears to be related to factors such as staffing, patient characteristics and attitudes that require further investigation.

## Data Availability

Raw data were generated at the University Department of Psychiatry and Psychotherapy Tuebingen and the Centers for Psychiatry Suedwuerttemberg. Some of the data are not publicly available due to their containing information that could compromise the privacy of research participants. Data that support the findings of this study are available from the corresponding author, LKS, upon reasonable request.
